# Reference Values and Related Factors for Peak Expiratory Flow in Middle-Aged and Elderly Chinese

**DOI:** 10.3389/fpubh.2021.706524

**Published:** 2021-08-20

**Authors:** Chao Ji, Yang Xia, Huixu Dai, Zhiying Zhao, Tiancong Liu, Shuhui Tong, Xiaohang Zhang, Yuhong Zhao

**Affiliations:** ^1^Department of Clinical Epidemiology, Shengjing Hospital of China Medical University, Shenyang, China; ^2^Department of Otorhinolaryngology—Head and Neck Surgery, Shengjing Hospital, China Medical University, Shenyang, China; ^3^Safety and Environment Protection Technology Supervision Center, Liaohe Oilfield Company, Panjin, China; ^4^Disease Prevention and Control Center of Shahekou District of Dalian City, Dalian, China

**Keywords:** elderly, household air pollution, peak expiratory flow, reference values, risk factors

## Abstract

**Background:** Peak expiratory flow (PEF), as an essential index used for screening and monitoring asthma, chronic obstructive pulmonary disease, and respiratory mortality especially in the elderly, is recommended for low-resource settings in low- and middle-income countries. However, few studies have focused on the reference of PEF in China, especially in middle-aged and elderly people. Thus, this study aimed to determine age- and sex-specific reference values of PEF in the middle-aged and elderly Chinese population.

**Methods:** There were 8,914 participants who were included for risk factor analysis and 5,498 participants included for reference value analysis. The PEF was measured using a peak flow meter in liters per minute. The distributions of standardized PEF terciles stratified by sex and age were reported. Multiple linear regression analysis was used to determine the associations between risk factors and PEF.

**Results:** The PEF was higher in men than women across all age subgroups. The value of PEF decreased with age in both men and women. Height, weight, handgrip strength, and residence in rural were positively associated with PEF. Age and smoking status were negatively associated with PEF significantly in both men and women (*P* < 0.05). The mean PEF values were 367.10 and 253.00 L/min for men and women, respectively. Meanwhile, the prevalence of low PEF was 3.94 and 3.32% for men and women, respectively.

**Conclusions:** Age- and sex-specific centiles of standardized PEF for the middle-aged and elderly Chinese population were estimated. The reference values for low PEF could provide reference standards for epidemiological studies and clinical practices in the future. Interventions to improve lung functions or to prevent respiratory disease should be paid more attention to factors associated with PEF.

## Introduction

Asthma and chronic obstructive pulmonary disease (COPD), as two of the most common obstructive airway diseases (1), are serious public health problems worldwide (2–4), especially in China (5–8). From 1990 to 2015, the prevalence of asthma and COPD globally increased by 44.2 and 12.6%, respectively (3). In 2015, disability-adjusted life years caused by COPD were 15,389,017 and 1,797,438 by asthma in China (3). Thus, it is important to seek a convenient, fast, and feasible method for early screening and disease surveillance. The peak flow meter is a handy, easy-to-use tool, which is convenient and cost-effective for investigation and personal care (9). Meanwhile, peak expiratory flow (PEF) was an essential index used to assess response to asthma treatment, short- or long-term monitoring of asthma (4, 9), or to evaluate triggers for worsening symptoms and screening of COPD (10). PEF is recommended by the WHO, especially for low-resource settings in low- and middle-income countries (11).

There are nearly 1.4 billion Chinese in the world, indicating a huge medical need. However, the research studies on the reference value of PEF are rarely reported. At present, reference values of spirometry for the Chinese population are calculated using southeast Asians and northeast Asians by adjusting with fixed ethnic conversion factors, which were mainly established with Caucasian data, and did not include PEF values (10, 12, 13). Moreover, several studies have shown that reference values for spirometry, derived from different ethnic populations, are not applicable to the population nationwide (13, 14). For example, a study of Chinese children aged 5–14 years suggested that the Greece and Ireland references were inappropriate for Chinese children (15).

Most previous studies in China have focused on children and adolescents (15, 16), with few reports on adults (10, 17), especially for middle-aged and elderly adults. Although one study (13) included 7,115 subjects aged 4–80 years in China, the middle-aged and elderly people accounted for only a small proportion, and relevant variables were not included. Another study based on the elderly was conducted in Jinan (17), a city of eastern China, which means that it could not represent the elderly population of the whole of China. However, there was still no nationwide reference value for Chinese people. The lack of nationwide PEF reference values made it hard to evaluate the PEF results accurately.

Previous studies showed that age was one of the most important determinants of predicted PEF, which was decreased with increasing age (9, 14). In addition, PEF was an independent predictor of health status, and physical and cognitive functions (18), hospitalization, frailty development (19), mortality from respiratory, and other causes in the elderly (19, 20). Household air pollution from inefficient cooking and heating of solid fuels discharges particulate matter and gases, which causes adverse health effects in human subjects (21), and is associated with the lung function of infants (22) and middle-aged adults (23). However, this association has not been explored in elderly adults.

Thus, this study aimed to investigate age- and sex-specific reference values of PEF in the middle-aged and elderly Chinese population based on the China Health and Retirement Longitudinal Study (CHARLS), which involves 17,708 middle-aged and elderly participants from 28 provinces in China. Moreover, we explored the factors associated with PEF, including household air pollution, which was rarely shown in the previous studies. Meanwhile, the sex-specific cutoff values were calculated and used to evaluate the results for PEF. That provided a criterion for chronic lung disease screening.

## Materials and Methods

### Study Population

**Figure 1:**
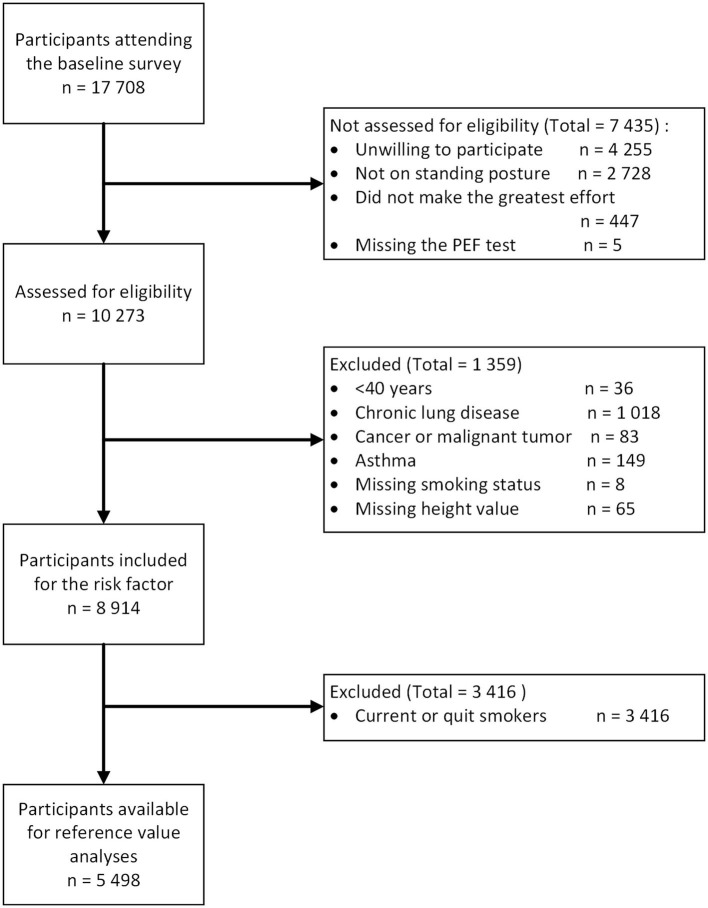
The flow chart of the peak expiratory flow (PEF) study.

The CHARLS is a longitudinal survey collecting data on demographics, socioeconomic status, biomedical measurements, and self-reported health status in China (24). This cross-sectional study used data from the baseline of CHARLS, which was conducted between June 2011 and March 2012. In total, 17,708 participants were included. The participants were selected using a four-stage, stratified, cluster random sampling method from 450 counties of 28 provinces, and the response rate was 80.5%. During the period of the research study, 10,273 participants performed the PEF test well. For this study, 36 participants younger than 40 years were excluded. Additionally, we excluded those who had chronic lung diseases (*n* = 1,018), cancer or malignant tumor (*n* = 83), asthma (*n* = 149), missing smoking status (*n* = 8), and height value (*n* = 65). COPD refers to a group of diseases that cause airflow blockage and breathing-related problems. It includes emphysema and chronic bronchitis, excluding tumors or cancer. Thus, 8,914 participants were included in PEF risk factor analysis. As 3,416 subjects were current smokers or who had quit smoking, only 5,498 participates were included in the reference analysis (Figure 1). The CHARLS was conducted at Peking University. The protocol was approved by the ethics committee of Peking University, and written informed consent was obtained from all participants in this study. The study protocol conformed to the ethical guidelines of the 1975 Declaration of Helsinki (25).

### Measurement of PEF

The PEF was measured using a peak flow meter (Everpure, Shanghai, China) in liters per minute. Each participant was asked to stand up, take a deep breath, place his or her lips around the disposable mouthpiece, and blow at maximum strength and speed. The trained technician recorded the readings indicated by the pointer, reset the flow meter, and then waited for 30 s before the next measurement. The measurement was repeated three times, and the maximum value was used in the analysis.

### Assessment of Other Variables

The demographic variables and health status functioning in this study were collected by standardized questionnaires that included sex, age, education level (no formal education, elementary school or middle school, and above), smoking status (current non-smoker or smoker), drinking status, place of residence (urban or rural), number of comorbidities, clean heating energy use, clean cooking fuel use, and clean energy use. Weight, height, and waist circumference were measured in the morning before breakfast. Participants wore light indoor clothes for weight measurement, using a health meter (Omron HN-286, Yangzhou, China) with an accuracy of 0.1 kg. Height was measured without shoes to the nearest 0.1 cm with a stadiometer (Seca 213, Hangzhou, China). Waist circumference was measured by a trained technician using an inelastic tape midway between the last rib and the top of the iliac crest on the midaxillary line. The body mass index (BMI) was calculated as weight in kilograms divided by square of height in meters (kg/m^2^). Handgrip strength was measured with a hand-held dynamometer (Yuejian WL-1000, Nantong, China) in kilograms (kg). Each hand was tested twice, alternating hands between tests, and the greatest force was used in the final analyses. Education level was attained through the question “What is the highest level of education completed?” “Illiterate” seemed as no formal education; “capable of reading or writing,” “Sishu/home school,” or “graduate from elementary school” seemed as elementary school; others were seemed as middle school and above. Smoking was defined as smoked more than 100 cigarettes in life. Drinking was defined as the respondent who has had any alcoholic beverages more than once a month. Information of comorbidities was collected through the question “Have you been diagnosed with conditions listed below by a doctor?,” and the options included the following: hypertension; dyslipidemia; diabetes or high blood sugar; liver disease; heart attack, coronary heart disease, angina, congestive heart failure, or other heart problems; stroke; kidney disease; stomach or other digestive diseases; emotional, nervous, or psychiatric problems; memory-related disease; and arthritis or rheumatism. The number of comorbidities means how many comorbidities they have. Household air pollution was assessed by a questionnaire. Clean heating status was assessed through the questions “Does your residence have heating?” and “What is the main heating energy source?” Neither heating nor heating source from solar, natural gas, liquefied petroleum gas, and electric was considered as clean heating energy use. Heating sources from coal and crop residue/wood burning were considered as solid fuel use. Source of cooking fuel from natural gas, marsh gas, liquefied petroleum gas, electric, or other was defined as clean cooking fuel use. Using both clean heating energy and clean cooking energy was defined as clean energy use.

### Statistical Analyses

Data were shown according to sex and age. The Kolmogorov–Smirnov test was used to evaluate the normal distribution of the variables. The continuous variables were reported as mean ± SD. Sex-specific reference equations for PEF were derived using the Box-Cox Cole and Green (BCCG) distribution, Lambda–Mu–Sigma (LMS) method implemented in the Generalized Additive Models for Location, Scale, and Shape (GAMLSS version 5.2-0) package (26) in the statistical software R (version 4.0.3, www.r-project.org). The PEF data were modeled with age and standing height as explanatory variables. Using BCCG distribution, the best model was estimated by exploring whether log transformations of dependent and independent variables were required. Considering the unnecessary over-fitting, the optimal degrees of freedom for the spline curve were chosen by selecting the model with the smallest Schwarz Bayesian Criterion. The goodness of fit was judged from inspection of normal Q–Q plots and worm plots. Sex- and age-specific centiles for PEF were predicted using the LMS method, which was standardized by height.

Multiple linear regression analysis was used to adjust for the effects of sex, age, weight, height, waist circumference, handgrip strength, education level, smoking status, drinking status, place of residence, number of comorbidities, clean heating energy source, clean cooking fuels use, and clean energy use and to identify those associated with PEF independently. Given the large population, and the relatively small number of predictor values, all the covariables were included in the regression model. Furthermore, a cutoff value was defined to identify persons with a “Low” PEF, which was expressed as 1 SD and 2 SD lower than the sex-specific mean value derived from people younger than 65 years old.

Data management and statistical analyses were conducted using SAS 9.3 edition (SAS Institute Inc., Cary, NC, USA). Estimation of percentiles was conducted using the GAMLSS package in the statistical software R. All reported *P*-values were two-sided, and *P* < 0.05 was considered significant.

## Results

### The Characters of Participants

A total of 8,914 observations of PEF from 17,708 participants were included in risk factor analysis. The characteristics of participants included in the reference analyses were shown in Table 1. The mean values of PEF were 367.10 ± 129.90 and 253.00 ± 94.76 L/min for men and women, respectively. Men had higher age, height, weight, PEF, the proportion of smoking and drinking status, and the number of comorbidities than women (*P* < 0.05). Compared with women, men had a lower level of BMI and lower education level (*P* < 0.05).

**Table 1:** Demographic characteristics of the participants.

**Variables**	**Total** **(*n =* 8,914)**	**Men** **(*n =* 4,152)**	**Women** **(*n* = 4,762)**
**Age** ^*^ **, years**	58.06 ± 9.41	58.77 ± 9.15	57.45 ± 9.86
**Height** ^*^ **, cm**	158.22 ± 8.56	164.23 ± 6.61	152.98 ± 6.33
**Weight** ^*^ **, kg**	59.11 ± 11.68	62.33 ± 11.57	56.31 ± 11.03
**BMI** ^*^ **, kg/m** ^**2**^	23.54 ± 3.88	23.03 ± 3.62	23.98 ± 4.03
**Waist circumference** ^*^ **, cm**	84.05 ± 12.51	83.84 ± 12.35	84.23 ± 12.64
**PEF** ^*^ **, L/min**	311.48 ± 123.88	367.10 ± 129.90	253.00 ± 94.76
**Handgrip strength, kg**	33.08 ± 11.97	39.77 ± 12.60	27.22 ± 7.50
**Education, %**			
No formal education	21.64	35.55	9.44
Elementary school	37.20	34.68	39.40
Middle school and above	41.16	29.77	51.16
**Smoking, %**			
Yes	31.02	59.96	5.68
No	68.98	40.04	94.32
**Drinking, %**			
Yes	25.02	45.57	7.02
No	74.98	54.43	92.98
**Place of residence, %**			
Urban	32.19	31.31	32.96
Rural	67.81	68.69	67.04
Number of comorbidities, median (Q1, Q3)	1 (0, 2)	1 (0, 2)	1 (0, 2)
**Clean heating energy, %**			
Yes	19.33	19.06	19.57
No	80.67	80.94	80.43
**Clean cooking fuel, %**			
Yes	44.41	43.88	44.87
No	55.59	56.12	55.13
**Clean energy use, %**			
Yes	12.77	12.53	12.98
No	87.23	87.47	87.02

* *Presented as means ± standard deviation. PEF, peak expiratory flow*.

### Factors Associated With PEF

The multiple stepwise linear regression associations between PEF and age, height, weight, waist circumference, handgrip strength, education level, smoking and drinking status, place of residence and number of comorbidities, clean heating energy use, clean cooking fuel use, and clean energy use among the middle-aged and elderly men and women in China are presented in Table 2. In both men and women, height, weight, handgrip strength, and residence in the rural were positively associated with PEF significantly. Age and smoking status were negatively associated with PEF significantly. Different from men, waist circumference, education level, the number of comorbidities, and clean energy use were associated with PEF in women.

**Table 2:** Multiple linear regression for PEF.

**Variables**	**Men**				**Women**			
	***β***	**SE**	***t***	***P***	***β***	**SE**	***t***	***P***
**Intercept**	208.07	58.23	3.57	<0.001	135.55	40.46	3.35	<0.001
**Age, years**	−3.83	0.24	−16.08	<0.001	−2.03	0.16	−12.67	<0.001
**Height, cm**	1.50	0.36	4.20	<0.001	1.06	0.26	4.09	<0.001
**Weight, kg**	1.68	0.26	6.39	<0.001	0.87	0.18	4.88	<0.001
**Waist circumference, cm**	−0.29	0.19	−1.48	0.139	−0.28	0.13	−2.16	0.031
**Handgrip strength, kg**	1.24	0.17	7.44	<0.001	1.63	0.20	8.26	<0.001
**Education level**								
No formal education	reference				reference			
Elementary school level	7.80	4.59	1.70	0.089	12.51	4.84	2.59	0.010
Middle school and above	5.30	4.89	1.08	0.279	13.69	4.79	2.86	0.004
**Smoking status**								
No								
Yes	−8.12	3.98	−2.04	0.041	−16.20	5.78	−2.80	0.005
**Drinking status**								
No	reference				reference			
Yes	−1.60	3.84	−0.42	0.676	−5.14	5.21	−0.99	0.324
**Place of residence**								
Urban	reference				reference			
Rural	12.78	4.41	2.90	0.004	6.78	3.04	2.23	0.026
Number of comorbidities	−1.77	1.72	−1.03	0.304	−3.34	1.12	−2.98	0.003
**Clean heating energy use**								
No	reference				reference			
Yes	10.35	8.05	1.29	0.199	−0.27	5.65	−0.05	0.962
**Clean cooking fuel use**								
No	reference				reference			
Yes	−5.98	4.43	−1.35	0.177	−5.06	3.12	−1.62	0.105
**Clean energy use**								
No	reference				reference			
Yes	14.69	10.09	1.46	0.146	16.89	7.07	2.39	0.017

*PEF, peak expiratory flow; SE, standard error*.

### Cutoff Values for Low PEF

A total of 7,036 participants aged ≤65 were enrolled to compute cutoff values for low PEF. The mean value of PEF was 389.02 ± 125.19 L/min for men and 275.79 ± 92.15 L/min for women in younger reference adults (≤65 years). Thus, as defined, at 1 SD below the sex-specific mean value, the cutoff values of low PEF were 263.83 and 183.64 L/min for men and women, respectively. Therefore, the prevalence of low PEF in men and women was 22.25 and 21.90%, respectively. When the reference was set as 2 SD below sex-specific mean value, the cutoff values of low PEF were 138.64 and 91.94 L/min for men and women, respectively. Meanwhile, the prevalence of low PEF was 3.94 and 3.32% for men and women, respectively.

### Reference Equations for PEF

A total of 5,498 non-smoking participants were included in the reference value analysis. The sex-specific reference equations and lookup tables for PEF are presented as files in the Supplementary Table 1. Age and height were nearly linearity related with PEF. The final equations were determined by the smallest Schwarz Bayesian Criterion value. Normal Q–Q plots and worm plots suggested a normal distribution and good fit.

The final equations were provided as follows:

PEF_for_ _male_=100.43–4.94^*^age (years)+3.47^*^height (cm)+Mspline
PEF_forfemale_=66.98–2.73^*^age (years)+2.31^*^height (cm)+Mspline

Mspline is the age- and sex-specific parameter in Supplementary Table 1. Lookup tables for PEF.xlsx.

### Age-Related Change in PEF

**Figure 2:**
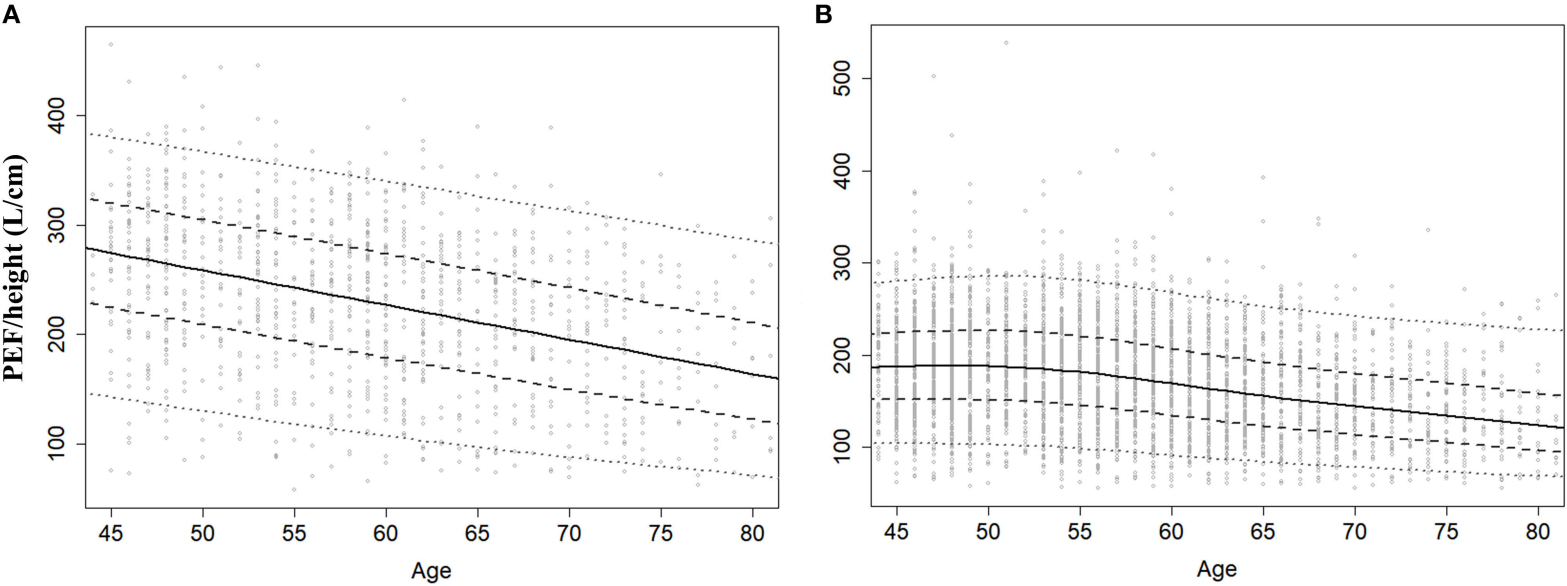
Centiles curve for standardized PEF men **(A)** and women **(B)**. Centiles were estimated by Lambda–Mu–Sigma (LMS) method.

Sex-specific percentiles 5, 25, 50, 75, and 95 for standardized PEF (PEF/height) were predicted using the BCCG distribution LMS method. The predicted percentiles of PEF had a downward trend with age for both men and women (Figure 2). Standardized PEF was higher among men than women at age 40–80 years. By the age of 40–50 years, the median of standardized PEF was nearly 1.5 times of women. With the increase in age, the ratio decreased to 1.1 at the age of 80 years and above.

## Discussion

In this study, we established the reference values for PEF using data from 5,498 healthy middle-aged and elderly Chinese adults according to age and sex. The mean values of PEF were 367.10 ± 129.90 and 253.00 ± 94.76 L/min for men and women, respectively. The PEF was higher in men than in women for all age subgroups. The PEF value decreased with age in both men and women. Our data also suggested that, in both men and women, age, height, weight, handgrip strength, residence in the rural, and smoking status were significantly associated with PEF. Besides, waist circumference, education level, the number of comorbidities, and clean energy use were associated with PEF only in women significantly. The cutoff values of low PEF (mean−2 SD) were 138.64 and 91.94 L/min for men and women, respectively, and the prevalence of low PEF in men and women was 3.94 and 3.32%, respectively.

As a physiologic measure and an important index, PEF has been proposed to estimate airflow obstruction, which could be used to help in the management of asthma (11) and screening of COPD (10). Compared with formal spirometry, which is usually unavailable in primary care settings, especially in developing countries (10), the PEF test is much cheaper and easier, although it is a crude measure. In addition, considering its rapid and easy-to-get results, it is also feasible for use in large-scale surveys (27). In recent years, some researchers found that PEF was not only related to respiratory disease but also demonstrated strong relationships with some poor outcomes (20, 27, 28), especially in elderly adults. Thus, the measurement of PEF has an important effect in clinical practice and practical significance in public health. Hence, it is essential and urgent to establish a unique reference for the Chinese.

Global Lung Function Initiative has recommended multiethnic spirometry reference values for Northeast Asians, which were established based on a large urban Caucasian data in 2012 (12). However, the reference value did not include PEF. Kodgule et al. (9) derived reference values for PEF based on Indian adults, which was not suitable for Chinese people. In a previous study, PEF reference values for Chinese were estimated. However, several disadvantages limited the nationwide usage, for example, based on children and adolescence, or small local regions, or small samples (15–17, 29–32). Jian et al. (13) estimated the reference value for 7,115 healthy Han nationality people aged 4–80 years. Parameters and equations for a low limit of normal for PEF in separate age segments were shown in their study. However, that is not convenient for clinical practice in screening people who have abnormal lung function. Moreover, potential factors associated with lung function were not analyzed adequately. Household air pollution, for example, has been found with respiratory disease, cardiovascular disease, and lung functions. The association between PEF and household air pollution has not been explored in the elderly. Therefore, our study was based on the CHARLS, which involves 17,708 middle-aged and elderly participants from 450 counties of 28 provinces in China, and cutoff points for men and women were estimated, respectively. These would make the clinical screening for patients who are abnormally very simple and practical.

There are some differences between our study and the previous study. Firstly, our study focuses on the middle-aged and elderly, including 8,914 Chinese people aged over 40 years. In the previous study, there was a wide range of age of 4–80 years for 7,115 subjects but the middle-aged and elderly accounted for only a small proportion (13). PEF is very important for the screening and treatment of asthma and COPD, especially for adults and elderly, those who were declined in lung function with age. Secondly, limitations in the previous study only included the limited factors influencing pulmonary function, such as age, sex, and height. However, in this study, we explored the relationship between PEF and age, height, weight, waist circumference, handgrip strength, education level, smoking and drinking status, place of residence, number of comorbidities, and clean energy use in addition to sex and age. Thirdly, PEF was measured using a peak flow meter, which has a somewhat higher intrasubject variability and a lower diagnostic accuracy compared with spirometry. But it can be a valid alternative as a first-line screening tool because of the advantages, such as it is inexpensive, portable, and quickly and easily used. The consistency of the PEF test by a peak flow meter and spirometer needs more studies to be confirmed.

Our data suggested that height, weight, handgrip strength, and residence in the rural were positively associated with PEF, and age and smoking were negatively associated with PEF significantly in both men and women. Besides, waist circumference, education level, the number of comorbidities, and clean energy use were associated with PEF only in women significantly. In addition, the cutoff point was defined as 2 SD below the mean value from subjects under the age of 65 years. The diagnostic criteria from fixed value are very practical in clinical practice (33).

There are several limitations that should be considered. First, this study population only comprised the middle-aged and elderly in China, which limits the generalizability of the results to other populations. Second, we did not consider the influence of some risk factors, such as nationality, environmental pollution, and physical activity, because such information was unavailable. Third, the impact of risk factors on PEF in this study was based on a cross-sectional study, which limits the inferences of causality.

## Conclusions

Peak expiratory flow declined with age from 40 years in both men and women. This study estimated the age- and sex-specific centiles of PEF for the middle-aged and elderly Chinese population using the LMS method. Interventions to improve lung function or respiratory disease should pay more attention to factors associated with PEF. More evidence is required from epidemiological studies and clinical practice in the future.

## Data Availability Statement

The original contributions presented in the study are included in the article/Supplementary Files, further inquiries can be directed to the corresponding author/s.

## Ethics Statement

The studies involving human participants were reviewed and approved by the ethics committee of Peking University. The patients/participants provided their written informed consent to participate in this study.

## Author Contributions

YZ: full access to all of the data in the study and took responsibility for the integrity of the data and the accuracy of the data analysis. CJ and YX: study concept and design. TL and XZ: acquisition of data. ST and ZZ: analysis and interpretation of data. CJ and HD: drafting of the manuscript. CJ and YX: statistical analysis. YZ: manuscript revision and critical comments and suggestions. All the authors read and approved the final manuscript.

## Conflict of Interest

The authors declare that the research was conducted in the absence of any commercial or financial relationships that could be construed as a potential conflict of interest.

## Publisher's Note

All claims expressed in this article are solely those of the authors and do not necessarily represent those of their affiliated organizations, or those of the publisher, the editors and the reviewers. Any product that may be evaluated in this article, or claim that may be made by its manufacturer, is not guaranteed or endorsed by the publisher.

## Abbreviations

PEF: Peak expiratory flow
COPD: chronic obstructive pulmonary disease
CHARLS: Chinese Health Retirement Longitudinal Study
BMI: body mass index
SD: standard deviation
BCCG: Box-Cox-Cole and Green
LMS: Lambda-Mu-Sigma
GAMLSS: Generalized Additive Models for Location Scale and Shape.
